# Immune Ageing Clocks: A Methods-Oriented Review of Tasks, Modalities, Models, and Recalibration

**DOI:** 10.3390/cells15050421

**Published:** 2026-02-27

**Authors:** Gengchen Yu, Zeyu Shao, Jingyu Zhuo, Zixuan Chen

**Affiliations:** Beijing Key Laboratory for Separation and Analysis in Biomedicine and Pharmaceuticals, School of Medical Technology, Beijing Institute of Technology, Beijing 100081, China; 3120231424@bit.edu.cn (G.Y.); 3220231970@bit.edu.cn (Z.S.); 3220242887@bit.edu.cn (J.Z.)

**Keywords:** immune ageing clock, immune ageing, age-type clock, risk-type clock, task lineage, multi-omics integration, external validation

## Abstract

Population ageing and the growing burden of immune-mediated disease have prompted efforts to quantify immunosenescence with clinically usable biomarkers. Immune ageing clocks have been built from immunophenotyping, transcriptomics, proteomics, epigenomics and adaptive receptor repertoires, but heterogeneous task definitions, assay protocols and evaluation criteria limit comparability and translation. We review major immune data modalities and outline an end-to-end workflow from cohort design and assay standardisation to preprocessing, feature engineering, model development, validation and recalibration. We propose a task–modality–model taxonomy separating (i) chronological age clocks, (ii) outcome-anchored risk clocks and (iii) cell lineage/state clocks, while treating bulk blood transcriptomics (whole blood or PBMC) as a molecular-layer modality that can support either age-scale or outcome-anchored tasks depending on supervision. Across studies, common limitations include batch effects, compositional confounding, endpoint mismatch, scarce external validation and limited mechanistic anchoring. We conclude with priorities for the field, including multimodal integration, longitudinal designs with digital phenotypes, tissue- and cell-type-specific models, and pathway-grounded clocks that can be linked to interventions.

## 1. Introduction

Rapid population ageing challenges global public health and imposes substantial economic costs [1]. Age is the dominant risk factor for chronic progressive disorders, including cardiovascular disease, cancer, metabolic disease and neurodegeneration [2,3]. At the mechanistic level, ageing reflects interacting hallmarks (e.g., genomic instability, telomere attrition and epigenetic drift with immune remodelling and changes in tissue microenvironments emerging as key contributors) [4,5,6].

Immunosenescence denotes the age-related remodelling of immunity that weakens primary responses to pathogens and vaccines and often co-occurs with chronic low-grade inflammation (“inflammaging”) [4,6]. In adaptive immunity, thymic involution reduces naïve T-cell output and skews pools toward memory/terminal differentiation, while B-cell ageing alters naïve-cell abundance and the efficiency of class switching and somatic hypermutation, affecting antibody quality and repertoire diversity [4,7,8]. Innate immunity also changes: neutrophils show impaired chemotaxis and phagocytosis, and natural killer (NK) cells display altered cytotoxicity and cytokine production, sometimes without large changes in cell counts [9,10,11]. Senescence-associated secretory programmes and tissue remodelling further reinforce inflammaging [6,12]. These features motivate integrative biomarkers that capture immune ageing at both population and individual levels [13,14,15].

Single biomarkers offer incomplete and often confounded views. Leukocyte telomere length (LTL) declines with age but varies widely and is sensitive to cell-mixture composition, inflammatory state and assay platform [16,17,18]. DNA methylation clocks (e.g., Horvath, Hannum, PhenoAge and GrimAge) track chronological age and predict morbidity and mortality, yet in whole blood they can be driven by compositional shifts rather than functional immune properties (e.g., activation or cytokine output) [19,20,21]. Reliance on one marker may therefore miss clinically relevant phenotypes such as impaired pathogen clearance, dysregulated inflammation, or treatment-related immune stress [18,22].

Accordingly, high-throughput immune profiling combined with statistical and machine-learning methods is increasingly used to build immune ageing (immunosenescence) clocks [13,14,23]. An immune ageing clock maps multidimensional immune features to an ageing-related score, enabling the estimation of immune age and deviation from chronological age (ΔImmuneAge). Inputs include cell composition and phenotypic states, soluble proteins, transcriptomic/epigenomic profiles and adaptive receptor repertoires [24,25,26]. Confounders such as clonal haematopoiesis of indeterminate potential (CHIP) can shift inflammatory baselines, whereas features such as CXCL9 illustrate how proteomic signals may connect clock outputs to clinical phenotypes [14,27,28]. Beyond chronological age prediction, clocks aim to capture functional variation, support risk stratification, prioritise mechanisms and enable longitudinal monitoring [21].

However, studies differ widely in task (age prediction, outcome/risk prediction, lineage-specific clocks), modality (cellular, molecular and clinical/digital context), modelling strategy (linear, tree-based, deep-learning, multimodal fusion) and evaluation practice (calibration, external validation, clinical utility) [29,30,31]. This heterogeneity limits comparison, replication and translation.

Prior reviews have summarised immunosenescence biology and ageing biomarkers, but often separate what is predicted (task), what is measured (modality) and how models are built and evaluated [4,32]. Here we integrate these axes into an actionable task–modality–model framework, emphasising deployment-focused evaluation (calibration, clinical net benefit and transportability/recalibration) [29,31,33]. We also synthesise recurrent failure modes—compositional confounding, batch/platform drift and weak external validity—and outline mitigation and reporting recommendations (Table 1).

**Figure 1:**
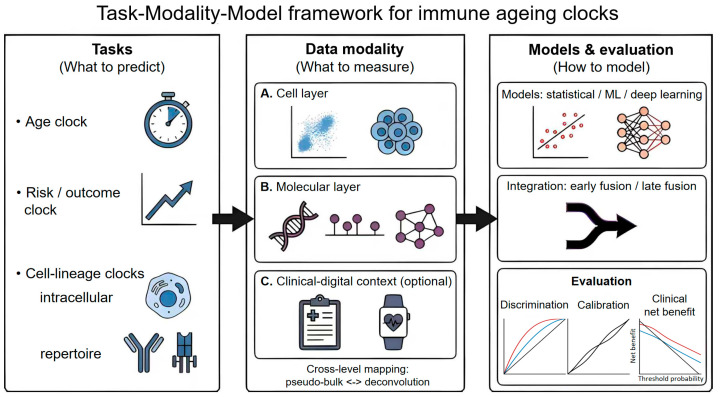
Task–modality–model framework for immune ageing clocks. We organize immune ageing clock studies along three complementary axes: tasks (age clocks, risk/outcome clocks, and cell-lineage clocks, including intracellular and repertoire-based clocks), data modalities (cell layer, molecular layer, and optional clinical–digital context), and models (statistical, machine-learning, and deep-learning approaches, with early or late fusion for multimodal integration). Cross-level mapping between cellular- and individual-level representations can be achieved via pseudo-bulk aggregation and pathway/module scoring, and via deconvolution to infer cell-type composition from bulk profiles. Model reporting should include discrimination, calibration, and clinical net benefit. Icon legend: the stopwatch denotes chronological age prediction; the upward trend arrow denotes risk/outcome prediction; the cell icon denotes intracellular (single-cell/cell-state) clocks; the antibody/TCR-like symbols denote immune repertoire-based clocks. In the modality panel, the scatter/cluster and cell-group icon represent cell-layer measurements (e.g., cytometry or single-cell profiles); the DNA helix, lollipop/marker symbols, and molecular network represent molecular-layer features (e.g., genomics/epigenomics/transcriptomics/proteomics and pathway/network summaries); the clipboard and wearable device represent clinical records and digital health context. In the model panel, the regression plot and neural network denote statistical/ML/deep-learning models, and the branching arrow denotes early/late fusion for multimodal integration. In the evaluation panel, the three mini-plots schematically represent discrimination, calibration, and decision-curve (clinical net benefit) assessment (colored curves illustrate example models and the black line a reference).

To provide an at-a-glance roadmap, we summarise the task–modality–model framework and its associated evaluation components in Figure 1.

## 2. Construction and Evaluation Methods for the Immune Ageing Clock

Converting immune ageing into a quantitative score depends less on selecting a single “best” algorithm than on establishing an end-to-end analytical workflow, including cohort design and follow-up, coverage of immune measurements across layers, standardised quality control and feature engineering, alignment of modelling objectives with target tasks, and appropriate evaluation and reporting criteria [60,61,62]. Each step can shift what a clock captures, ranging from age-associated immune remodelling to broader inflammatory burden and its potential clinical implications [14,63].

**Figure 2:**
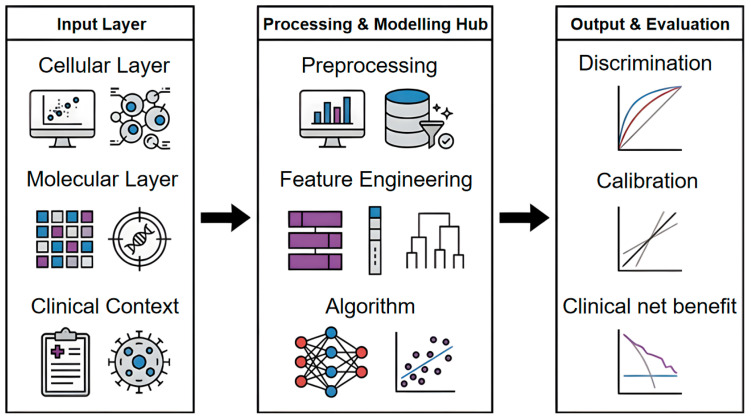
The input layer integrates cellular-level data (e.g., single-cell transcriptomics, cytometry, T-cell receptor (TCR)/B-cell receptor (BCR) repertoires), molecular-level readouts (e.g., bulk transcriptomics, proteomics, DNA methylation and other omics), and clinical context including demographics, exposures, comorbidities, and outcomes. In the processing and modelling hub, data undergo quality control and preprocessing, followed by feature engineering (e.g., feature selection, pathway/network aggregation, and latent representations) and model fitting using statistical or machine-learning algorithms. The output and evaluation block summarizes model performance in terms of discrimination (e.g., ROC curve, AUC, C-index), calibration, and clinical net benefit (e.g., decision-curve analysis). Icon legend: the monitor and cell symbols denote cellular profiling; the grid, target/DNA symbol denote molecular assays; the clipboard represents clinical data. Bar blocks and dendrograms indicate feature engineering; the neural network and scatter plot represent modelling algorithms. Curves in the evaluation panel illustrate example model performance and reference baselines.

Accordingly, immune ageing clocks are better viewed as a methodological framework rather than a standalone model [60]. Here we outline the workflow from data selection to evaluation. We organise inputs by their analytical resolution (e.g., cell-layer measurements versus molecular-layer readouts) and summarise common preprocessing and feature construction procedures that yield robust representations. We then compare modelling options across linear/survival models, tree-based methods, kernel approaches, and deep neural networks, and integrate complementary evaluation dimensions spanning discrimination, calibration and clinical utility, with an emphasis on transparent reporting and reproducibility [29,31,55]. An overview of the end-to-end methodological pipeline is shown in Figure 2.

### 2.1. Core Data Types and Sources

#### 2.1.1. Analytical Units and Cross-Level Mapping

With respect to data dimensionality, we emphasise scale and analytical unit (cell layer versus individual/tissue layer) rather than the molecular species measured. Cell-layer data preserve lineage/state information at a single-cell or cell-subset resolution, whereas molecular-layer data capture omics and circulating signals at the level of an individual or tissue [32,64]. These layers are not mutually exclusive: single-cell profiles can be aggregated to the individual level (e.g., pseudo-bulk and pathway/module scoring), while bulk profiles can be decomposed to estimate cellular composition, enabling cross-level alignment for fusion modelling [36,47,65].

#### 2.1.2. Cellular Modalities

Cell-layer inputs are commonly obtained from whole blood, peripheral blood mononuclear cells (PBMCs), or tissue-derived single-cell suspensions. Classical immunophenotyping (flow or mass cytometry) summarises cell-subset proportions and activation/suppression phenotypes, capturing compositional and homeostatic shifts at the individual level [66,67]. Single-cell multi-omics (e.g., single-cell RNA sequencing (scRNA-seq), cellular indexing of transcriptomes and epitopes by sequencing (CITE-seq), and single-cell assay for transposase-accessible chromatin using sequencing (scATAC-seq)) resolves finer lineage and state variation; it can be summarised into an “individual × lineage/subpopulation” feature matrix for system-level clocks while also supporting within-lineage state-drift modelling (“intracellular age clocks”) [37,38,68]. Adaptive immune receptor repertoire (AIRR) sequencing (AIRR-seq) (bulk or single-cell repertoires) provides clonotype-level metrics such as diversity, clonal expansion, and public clones, which quantify immune experience and responsiveness and therefore support disease- or vaccine-related clocks [35,69,70].

#### 2.1.3. Molecular Modalities

Molecular-layer samples are typically collected at the individual/tissue level, including bulk transcriptomics (whole blood/PBMCs), epigenomics (leukocyte DNA methylation), circulating proteins/soluble factors (plasma/serum), and metabolomics/lipidomics (plasma/serum and other body fluids) [19,71,72]. Bulk transcriptomics is often condensed into stable pathway/module scores that support age- or risk-oriented clocks as standalone modalities [71,73]. DNA methylation underpins epigenetic age clocks and can serve as a baseline comparator (age gap estimation) and a source of candidate CpG features; regulatory annotations can further support mechanistic interpretation [19,20,74,75,76]. Proteomics and soluble factors reflect inflammatory and secretory states and are widely used for protein/inflammation-based risk clocks [14,77]. Metabolomics and lipidomics capture systemic metabolic contexts that can form independent clocks or complement multimodal models for outcome prediction [72,78,79,80].

Overall, molecular layers provide pathway- and microenvironment-level constraints, whereas cellular layers provide compositional and lineage/state interpretations; integrating both can improve interpretability and generalisation beyond single-modality clocks.

### 2.2. Modelling Approaches

Immune ageing clock studies commonly use both classical statistical models and more flexible machine-learning/deep-learning approaches. The regression-based framework provides a structured baseline for benchmarking results and for integration with established risk factor frameworks, whereas deep-learning offers additional capacity for high-dimensional modelling, nonlinear learning, and cross-modal integration [44,61].

Statistical modelling complements machine-learning by providing interpretable baselines and principled uncertainty. Linear and regularised regression (ridge, lasso, elastic net) is common for omics clocks because it handles p ≫ n settings and yields stable signatures. Generalised linear models extend this to binary or count outcomes, while Cox and other survival models support time-to-event endpoints and facilitate comparison with conventional risk scores [19,81]. Mixed-effects and longitudinal models can incorporate repeated measures, separating within-person change from between-person differences. Many studies train an age predictor and use age acceleration (predicted − chronological age) as an explanatory variable in downstream regression or survival analyses, reporting effect sizes and confidence intervals [43,82,83].

Tree-based ensembles (random forests, gradient boosting such as XGBoost/LightGBM), kernel methods and deep networks can capture nonlinear immune–age relationships and facilitate multimodal integration. Ensembles impose few distributional assumptions and scale well to proteomic and transcriptomic data; interpretability is often added via feature importance and SHAP [42,47,80]. Support vector machines (SVMs) can be competitive when sample sizes are moderate but feature spaces are high-dimensional, as is common for immunophenotyping panels. Deep-learning is most advantageous in large-cohort or pretrained settings, where it can learn shared latent representations for multi-omics inputs and longitudinal trajectories. For instance, iAge has been described as a deep-learning-derived inflammatory risk-relevant score (often reported on an age-like axis) and is associated with frailty, multimorbidity and vascular ageing; model attribution highlighted candidate drivers including CXCL9, linking the score to endothelial dysfunction [14,33,41,44,61].

### 2.3. Workflow Standardisation

#### 2.3.1. Task Definition and Cohort Design

An immune ageing clock typically follows three stages: (i) task definition and cohort design, (ii) preprocessing/feature construction and model training, and (iii) evaluation, validation and deployment-oriented updating. Early decisions—target endpoint, sampling strategy, assay protocol and covariate collection—often dominate downstream performance [56,61]. Later steps should explicitly address technical variation (batch/platform drift), biological confounding (cell-mixture effects, infection status, medication use) and transportability (external validation and recalibration) [30].

#### 2.3.2. Preprocessing and Feature Engineering

After defining the task and cohort, preprocessing and feature construction should maximise the biological signal while suppressing technical variation. Harmonisation across centres, platforms and sample-processing steps is essential. Otherwise batch effects and QC artefacts can masquerade as age signals and undermine external validity [84,85,86]. For flow and mass cytometry, this includes consistent panels, compensation, gating definitions and drift control (e.g., bead standards), followed by normalisation or batch correction when required [66,87]. Bulk transcriptomic/proteomic assays similarly need sample-level QC, the filtering of low-signal features and explicit assessment/correction of centre- and platform effects (e.g., ComBat/SVA-style approaches) [84,85]. Single-cell data add cell-level QC (doublet removal, mitochondrial/ambient RNA filtering) and integration methods that preserve lineage structure [36,45,46,88]. Feature engineering improves transferability by converting raw high-dimensional data into robust summaries, including cell-type proportions and ratios (e.g., naïve:memory) [65,73,89], pathway/module scores, repertoire diversity and clonal-expansion metrics, or pseudo-bulk/latent embeddings for single-cell and longitudinal trajectories [36,47,80]. Where bulk assays are used, deconvolution against single-cell references can partially separate cell-mixture shifts from cell-intrinsic ageing signals [22,65,90,91]. The goal is to use representations that reflect immune structure and function, improve interpretability and generalise across cohorts [32,79].

#### 2.3.3. Validation, Recalibration and Reproducible Release

With a stable feature set, model development should couple training with internal validation and explicit recalibration to support external use [30,61]. Interpretable baselines (regularised regression, survival models, shallow trees) should be reported alongside more complex kernels or deep networks, which need justification based on sample size, modality and the intended endpoint [41,62]. Internal validation (held-out test sets or nested cross-validation) should report age-fit metrics (MAE, RMSE, R^2^) and, for outcome clocks, discrimination metrics (C-index, time-dependent AUC) with confidence intervals [92,93]. When outputs are probabilistic, calibration should be assessed and corrected [29,94]; when outputs are age-like scores, ΔAge can be evaluated for incremental value beyond chronological age and conventional risk factors [21,82]. Transportability requires external validation across sites, platforms and age distributions, and the recalibration of intercepts/slopes or baseline hazards when population or platform shifts are present [30,95]. Analyses should pre-specify splitting strategies to avoid leakage across batches or repeated measures [33,50]. Reproducibility should be treated as a deliverable: share de-identified feature matrices, model weights/hyperparameters and minimal code, and provide scripts for external validation and recalibration [55,57,58]. Reporting can follow TRIPOD/TRIPOD-AI and related AI reporting checklists, while bias/applicability appraisal can use PROBAST/PROBAST + AI; community standards (e.g., MIFlowCyt, MiAIRR) improve metadata completeness and reuse [66,70].

### 2.4. Evaluation Framework for Immune Ageing Clocks

#### 2.4.1. Discrimination and Model Fit

For time-to-event outcomes (e.g., mortality, hospitalisation, and severe infection), model discrimination is commonly assessed using the concordance index (C-index) [92,96]. Harrell’s C-index is commonly used; however, it can be biassed under heavy censoring or heterogeneous follow-up times, whereas Uno’s C-index is more robust in these settings [92,93]. Time-dependent receiver operating characteristic (ROC) curves and the corresponding area under the curve (AUC) evaluate discrimination at pre-specified horizons (e.g., 1-, 3-, and 5-year risk) [92,93]. For chronological age prediction, mean absolute error (MAE) and root mean squared error (RMSE) quantify error, while correlation (or R^2^) summarises overall fit to chronological age [61,62]. More importantly, the association between ΔImmuneAge and outcomes should be quantified using survival models or generalised linear models (e.g., hazard ratio per 1-year increase in ΔImmuneAge with a 95% confidence interval) and tested for consistency across predefined strata [21,82,97].

#### 2.4.2. Calibration and Probability Accuracy

Calibration assesses whether model outputs correspond to observed risk or age on an absolute scale [29,94]. For probabilistic predictions, calibration-in-the-large and the calibration slope diagnose systematic over- or underestimation; reliability diagrams and Brier scores summarise overall accuracy [94,98]. For time-to-event models, calibration can be evaluated at fixed horizons with observed–expected plots and integrated calibration indices [92,96]. For age clocks, the analogue is agreement between predicted and chronological age across the age range and subgroups; reporting both the intercept and slope helps distinguish bias from noise [19,83]. When miscalibration is present, post hoc updates (intercept/slope adjustment, isotonic regression, Platt scaling) can improve portability without retraining [61,95,98].

#### 2.4.3. Clinical Utility and External Validity

High discrimination does not guarantee clinical usefulness [29,96]. Utility depends on decision thresholds, prevalence and downstream actions. Decision-curve analysis (DCA) estimates the net benefit across plausible thresholds and can compare a clock to treat-all/treat-none strategies or to standard risk scores [31,99]. Net reclassification improvement (NRI) and related metrics can quantify changes in risk strata, but should be accompanied by absolute risk changes and calibration [100]. For immune ageing clocks, candidate use cases include identifying vaccine non-responders, selecting older adults for enhanced surveillance, or monitoring the response to interventions (e.g., caloric restriction, mTOR inhibition) [101,102]. Such applications require pre-specified endpoints, external validation and clear reporting of what the clock adds beyond age and established clinical predictors [30,103,104].

## 3. Task Taxonomy of Immune Ageing Clocks

Current immunosenescence clocks are heterogeneous, differing substantially in input data types and modelling choices [14,23,73,97]. Inputs range from high-dimensional immune phenotypes to PBMC transcriptomes, peripheral proteomes, adaptive receptor repertoires, and single-cell epigenetic features [71,77,89,105]. Grouping clocks only by modality (e.g., PBMC, proteomics, or T-cell-focused readouts) obscures whether they address comparable questions or target different objectives [14,21]. A more practical approach is to classify clocks by prediction task—chronological age, clinical risk/outcomes, or cell/lineage-level clocks that encode time in cellular states or clonal structure [19,68,82]. Whole blood transcriptional age can serve as a systems-level reference to position immune ageing clocks within the task–modality–model framework. Which question does each clock answer best, and how do they complement each other across settings [24,71]? Key representative clocks and their methodological profiles are given in Table 2.

### 3.1. Calendar Age-Driven Age-Clock Immune Chronology

Chronological age-supervised immune ageing clocks are calibrated to the chronological age scale; ΔImmuneAge (predicted minus chronological age) is interpreted as relative acceleration or deceleration versus age-matched peers [19,74]. Existing studies largely follow two trajectories: (i) system-level clocks based on immune phenotypes and PBMC transcriptomes and (ii) humoral output-oriented clocks leveraging plasma proteomics and IgG glycomics [13,14,104].

At the system level driven by immune phenotypes and PBMC transcriptomes, the seminal IMM-AGE study started with high-dimensional immune phenotypes collected over time [13]. The approach defined a stable immune age trajectory and translated it into reusable functional descriptors linked to gene-regulatory activity [13]. Its independent prognostic value for all-cause mortality was then evaluated in an external population cohort (e.g., the Framingham cohort) [13]. Key methodological features include explicit task definition, survival-informed evaluation using ΔIMM-AGE in outcome-association analyses, expression signature representations for scalable immune phenotypes, and regularised linear models within a Cox framework to prioritise interpretability and external validity [13].

At the PBMC bulk transcriptome level, many immune-related genes were systematically associated with age [71,83]. Module-based approaches have enabled stable immune transcriptional age clocks (median absolute error ~3–5 years) and suggested midlife transcriptional inflection points [24,71]. With higher-quality PBMC single-cell data, single-cell immune clocks can resolve ageing signals at the subpopulation level [23,73]. Across scRNA-seq age gradients, age-associated signals often concentrate on a limited set of subsets, including specific NK states, CD8+ memory T-cells, and CD14+ monocytes [63,109]. This signal reflects both shifts in their relative abundance and changes in their cellular state [105]. Reports of immunological age acceleration in infectious and autoimmune diseases provide a cell–biological interpretation of “faster ageing” inferred from these clocks [23,101].

In contrast, humoral molecular clocks leverage circulating molecular networks derived from plasma proteins and IgG glycosylation [107]. Proteomic age clocks trained on large cohorts (e.g., UK Biobank) show strong chronological age prediction and generalise across independent cohorts [24,77]. Although trained on chronological age, proteomic age acceleration has been associated with chronic disease burden and all-cause mortality across multiple cohorts, supporting its use as a risk-relevant marker [14,77]. Gradient-boosted trees are commonly used to model high-dimensional proteomic features [77,78]. In survival analyses, ΔAge can be linked to outcomes (e.g., hazard ratios), but its definition and calibration should be specified and, when needed, re-estimated to ensure cross-cohort comparability [21,82]. IgG glycomics clocks (e.g., GlycanAge) capture age-associated shifts in Fc N-glycan profiles and yield ΔAge measures associated with metabolic and inflammatory phenotypes, including changes after lifestyle interventions [104,107].

In general, calendar age-supervised immune age clocks address two related questions. One infers immune age from peripheral blood immune composition/state, whereas the other reflects immune ageing via circulating molecular readouts [13,73]. The former is well suited to the mechanistic interpretation of immune function and its responses to infection or vaccination, whereas the latter supports rapid, high-dimensional profiling across large cohorts [63,109]. Both are constrained by peripheral blood sampling and may miss tissue-resident immune ageing [5].

### 3.2. Risk Clock-Type Immune Clocks with Mortality and Multimorbidity as Endpoints

Risk clocks are trained directly on clinical endpoints (e.g., mortality, frailty, multimorbidity), rather than chronological age. [14,21]. Accordingly, their outputs should be interpreted as an endpoint-anchored risk score (or predicted risk/probability) rather than an “age gap”. Age gap language is appropriate only for chronological age-supervised models, unless a risk score is explicitly recalibrated to an age-like scale with clear assumptions and external validation [32,60].

iAge exemplifies an inflammation network risk clock. Using inflammatory/immune proteomic panels from participants aged 8–96 years, deep models estimate an “inflammatory age” that associates with polypharmacy, frailty and vascular ageing, and predicts cardiovascular events and mortality. Attribution analyses highlighted CXCL9; follow-up experimental work linked CXCL9 signalling to endothelial dysfunction and arterial stiffness, illustrating a clock → pathway → mechanism workflow [14]. Related proteomic studies often train an age clock and then analyse age acceleration in survival models, or directly supervise on clinical endpoints. For risk clocks, evaluation should emphasise discrimination and clinical utility (C-index, time-dependent AUC, decision-curve analysis) and interpret ΔAge cautiously: it may reflect a risk continuum rather than the pace of ageing [93,96,99].

### 3.3. Cell/Lineage Clocks Based on Cellular States and Adaptive Receptor Repertoires

Compared with age and risk clocks, cell/lineage clocks emphasise where “time” is encoded in the immune system—i.e., which lineages/clones carry ageing-associated signals and how these signals are stored in epigenetic states or receptor repertoires at the cellular resolution. These clocks help localise how immune ageing manifests within specific lineages and clones [23,63,73].

Across T-cell lineages, multi-omics studies consistently report fewer naïve T-cells and a relative expansion of memory and terminally differentiated populations with age [109,110]. Across tissues, PD-1^+^TOX^+^CD8^+^ exhaustion-like subsets can be prominent and are often linked to chronic local inflammation and impaired metabolic homeostasis [63,109]. Consistent with these observations, single-cell PBMC-based immune clocks also highlight specific age-associated subsets, including NKG2C^+^GZMB^+^XCL1^+^ CD8^+^ memory T-cells, as being robustly age-sensitive across cohorts [109].

The TCR/BCR repertoire-driven “repertoire structure” clock focuses on the diversity and clonal expansion pattern of the adaptive immune repertoire [69,89]. Older adults typically show reduced diversity, expanded dominant clones, and shifts in public clonotypes [35,111]. By clustering similar sequences into functional units, the diversity and expansion of these units can be mapped to age or disease states, enabling repertoire-based age or risk scales. In addition, metadata standards such as MiAIRR facilitate cross-cohort harmonisation and external validation by enabling interoperable repertoire annotations [70,105,111]; this, in turn, supports integrative analyses linking immune exposures, repertoire architecture, and downstream clinical outcomes.

Cell and lineage clocks are valuable because they enable the decomposition of aggregate system-level readouts into interpretable cellular components [105]. Such decomposition can enable investigators to quantify accelerated immune ageing, identify the lineages that contribute most strongly, and, where applicable, infer division histories for expanding clones—thereby providing a foundation for lineage-targeted interventions and tissue-specific mechanistic studies [63,103,112].

## 4. Current Challenges

**Figure 3:**
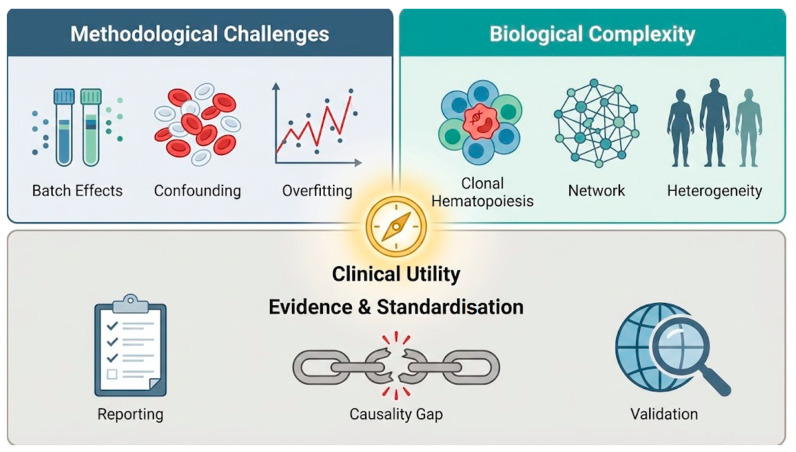
Major barriers hindering the clinical translation of immunosenescence clocks. The diagram presents a tripartite framework of challenges that limit robust clinical utility (central compass hub). Methodological challenges (top left) include batch effects across platforms, confounding driven by shifts in cell composition, and limited generalizability due to model overfitting. Biological complexity (top right) reflects intrinsic biological factors such as clonal hematopoiesis, interconnected inflammation–metabolic networks, and substantial inter-individual heterogeneity. Evidence and standardization gaps (bottom) highlight insufficient adherence to reporting guidelines (e.g., TRIPOD), the gap between statistical association and mechanistic causality, and limited external validation across diverse populations. Together, these barriers represent key bottlenecks that must be addressed to advance immunosenescence clocks from exploratory research to reliable clinical tools.Icon legend: laboratory tubes and cell symbols denote technical variability and cellular confounding; the upward trend plot indicates overfitting; the clonal cell cluster, network diagram, and human silhouettes represent biological complexity and heterogeneity; the clipboard, broken chain, and globe/magnifier denote reporting standards, causality gaps, and external validation, respectively; the central compass symbolizes clinical utility as the guiding objective.

Recent studies outline a workable framework for immune ageing clocks spanning task definition, modality selection, modelling and evaluation [32,60,61]. Blood-based markers can be derived reproducibly and linked to morbidity and survival, but transportability to diverse real-world settings remains the key barrier [21,77,82]. Methodologically, high-dimensional immune profiling amplifies batch/platform effects, compositional confounding and the sensitivity of nonlinear models to feature selection and data splits [22,41,113]. Biologically, immune ageing is heterogeneous and shaped by genetics, sex, chronic inflammation and clonal haematopoiesis, complicating any single scalar score. Finally, inconsistent reporting and largely correlational evidence limit clinical actionability [102,114]. Figure 3 summarises these barriers.

### 4.1. Methodological Challenges: Batch Effects, Compositional Confounding and Cross-Cohort Transportability

First, batch effects and inter-platform variability are unavoidable. The immune ageing clock is mostly based on multi-omics data from different cohorts and platforms. Flow cytometry and mass cytometry data are often generated across laboratories using different antibody panels, whereas transcriptomic and proteomic assays may rely on heterogeneous sequencing platforms or mass spectrometry-based workflows [67,115,116]. Differences in sampling time, pre-analytical handling, processing protocols, and cryopreservation conditions can introduce systematic biases [85,116]. Batch correction methods such as ComBat and Harmony (and related variants) can attenuate obvious batch-driven clustering to some extent [46,84,117]. However, their performance still depends on the covariates specified and the effective sample size. Omitting key covariates, or misclassifying true age-related biology as technical batch effects, can leave residual spurious differences or over-correct and attenuate genuine signals [85,116]. Such uncertainty may be masked during model development but can manifest as poor transportability during external application [30,33,50].

Cell composition confounding often co-occurs with batch effects. Whole-blood and PBMC bulk omics clocks are intrinsically mixture models, capturing both cell-intrinsic molecular drift and shifts in subpopulation proportions [90,118]. Deconvolution can help, but performance degrades for rare subsets, acute inflammatory perturbations or therapy-induced remodelling [91,108]. Consequently, clock outputs during infection or cancer treatment may reflect transient immune activation rather than underlying ageing. Although nonlinear models can capture complex structure, they are sensitive to data splits and feature selection choices [55,61]. Many studies report a favourable internal error or C-index but lack independent multi-site validation; stratified external validation and recalibration remain uncommon [29,30].

Finally, even beyond statistical performance, cross-clock comparability remains limited. Clocks such as IMM-AGE, iAge, proteomic age clocks, and IgG glycomics-based clocks can be applied to the same individuals but capture different immune dimensions [13,14,15,77,107]. Consequently, the direction and magnitude of “acceleration” can disagree across clocks [81]. The absence of a commonly recognised framework for comparing the “complementarity” and “redundancy” of these clocks toward a specific task, with control over model complexity and input modality variations, also hinders their parallel interpretation in real-world applications [55,96,119]. To facilitate parallel interpretation across clocks, the major sources of cross-cohort heterogeneity and recommended mitigation tools are summarised in Table 3.

### 4.2. Biological Challenges: Heterogeneity, CHIP and Inflammation–Metabolism Networks

Methodological challenges largely concern measurement and analytical choices, whereas biological challenges reflect the intrinsic complexity of the underlying immune system. Ageing of the immune system is affected by the genetic background, gender, hormones, long-term infections such as CMV, lifestyle, and environment [15,123,124]. Immune profiles between people of the same age can vary as much as those who are ten years apart or more [23,110]. Clocks derived largely from single time-point sampling often cannot disentangle the contributions of a lifelong elevated inflammatory baseline, recent infectious exposures, and short-term stress responses to the observed readouts. Without longitudinal trajectories and mechanistic anchors, “accelerated immune ageing” may be interpreted as a composite indicator rather than a specific modifiable pathway [106,125].

Clonal haematopoiesis of indeterminate potential (CHIP) provides a salient example. With age, haematopoietic clones carrying recurrent driver mutations (e.g., DNMT3A and TET2) can undergo preferential expansion, gradually altering blood cell composition and contributing to chronic, low-grade inflammation [27,28]. CHIP raises both the danger of hematologic malignancies and the chance of having atherosclerosis and heart problems. Across multiple cohorts of older adults, this signal can emerge as a latent layer that is not directly observable from single measurements but becomes apparent through integrative modelling [27,28]. If CHIP is not found or distinguished during clock-building analysis, then the age signal learned by the model probably corresponds to fitting CHIP status instead of more general immune ageing. It creates a source of systematic bias for subsequent clocks planned for extensive use among elderly populations [27,28].

Inflammation, endocrine regulation, and metabolism are tightly coupled during ageing, complicating the separation of immune age signals from cardiometabolic dysfunction, sarcopenia, and cognitive decline [6,72]. Clocks dominated by inflammatory and proteomic signals often show strong associations with polypharmacy and mortality risk, but may partly capture systemic frailty and immunometabolic dysregulation rather than immune-specific dysfunction alone. However, it is often unclear how much of ΔAge reflects immune-specific dysfunction versus broader systemic frailty and metabolic dysregulation. The clinical meaning of an elevated score can therefore be context-dependent across diseases and treatment settings, underscoring the need for task-specific validation and calibration [14,28].

### 4.3. Evidence and Standards: From Correlational Labels to Actionable Metrics

Asymmetry between evidence levels and reporting standards is another barrier to the clinical translation and broader acceptance of immune ageing clocks. Most evidence to date is derived from retrospective analyses or the secondary modelling of datasets originally generated for other purposes. These studies link immune age gaps to outcomes such as mortality, frailty, or multimorbidity, but conclusions are often cohort- and endpoint-specific. While informative for hypothesis generation, such associations do not automatically yield an actionable clinical metric. Clinical or public health adoption requires multicentre prospective studies with pre-specified clock endpoints and decision thresholds—evidence that remains scarce [59,126,127].

The reporting quality also varies substantially. Data splits are not always independent, feature selection and hyperparameter tuning may not be confined to training data (risking leakage), and “external validation” is sometimes performed on cohorts with only minor distributional shifts; these details are often underreported [33,57]. Model calibration status, threshold selection rationale, decision-curve analysis, and potential clinical net benefit assessments are often missing or only briefly discussed in appendices. Without standardised reporting, immune ageing clocks are difficult to reproduce and to incorporate into guidelines or consensus statements [55,56].

Interpretation and communication pose additional risks. Phrases such as “immune age is 5 years older than peers” may be technically valid as a model- and population-specific relative position, yet non-specialists may interpret it as definitive biological acceleration. Communicating such readouts without intervention evidence may increase anxiety and encourage unnecessary testing or treatment escalation. On the other hand, if there is evidence of intervention, we need to be careful not to use one clock reading as the sole basis for making decisions. These issues are rarely discussed explicitly but are essential for responsible real-world deployment [59,127].

In summary, from the perspective of methodology, biology, and evidence, the current immune ageing clocks are still in a state of “fast growth but no convergence”. Addressing these limitations—and tempering the interpretation of clock outputs—will be necessary to move beyond cohort- and model-specific optima. This development will take us closer to having a real, comparable, repeatable, understandable number system for measuring how old your immune system is.

## 5. Future Outlook

In the next phase, the emphasis may shift from further incremental gains in predictive accuracy to improving clinical and translational utility, moving immune ageing clocks beyond methodological demonstrations toward practical tools that support multiple downstream applications. Deep multi-omics integration and immune digital twin frameworks aim to establish mechanistically grounded and interpretable representations of immune ageing [128]. In parallel, tissue- and cell-type-specific clocks can help localise where ageing-associated changes arise and which lineages exhibit the earliest or most pronounced alterations; coupling clock outputs to testable causal pathways may further enable intervention-oriented evaluation and support the development of cross-cohort- and cross-platform-comparable immune age scales. Together, these directions outline how immune ageing clocks may evolve and mature as a translational field.

### 5.1. Multimodal Integration and Deep Models

Emerging multimodal models integrate immune-cell phenotypes, molecular profiles and contextual clinical data. Joint embedding approaches (e.g., totalVI, MOFA+) can align modalities and mitigate batch effects, while foundation-model strategies (e.g., scGPT) aim to learn transferable representations from large single-cell corpora [44,47]. These methods may enable clocks that generalise across platforms and capture lineage-specific ageing states, but they increase the risk of overfitting and therefore require transparent reporting, external validation and careful ablation to show what each modality contributes [57].

Multi-timescale integration is also required to align measurements collected at different temporal resolutions. For example, continuous digital biomarkers from wearables (heart rate variability (HRV), sleep microstructure, activity, and respiratory patterns) can be integrated with low-cost, low-burden humoral panels (e.g., C-reactive protein (CRP), interleukin-6 (IL-6), and selected chemokines) to shift immune burden assessment from sporadic blood draws toward longitudinal monitoring [40]. Because HRV and related physiological signals are associated with inflammatory states, wearable-derived trajectories may help detect early deviations consistent with infection or recovery dynamics and support population-scale surveillance, while remaining interpretable at the individual level. Under such a framework, immune ageing estimates could be modelled across slower baseline drifts (months to years) and faster fluctuations (days to weeks), enabling a coherent hierarchy of temporal dynamics [40,125].

At an individual level, the immune digital twin serves as a container for all this information [128]. This kind of hybrid twin is driven by multi-omics and clinical–physiological data, mechanistic models at key places, which might be able to simulate different vaccination regimens, immunomodulatory medicines, or lifestyle changes’ impact on immune routes and clock figures in a simulated setting. These simulations could support individualised hypothesis testing and intervention prioritisation. International immunological digital twin blueprints and early prototypes suggest the feasibility of representing immune age as a state variable, rather than a single scalar output, within digital twin frameworks [128].

### 5.2. Tissue- and Cell-Type-Specific Clocks

Most existing immune ageing clocks are built from peripheral blood, implicitly assuming that blood-derived readouts approximate systemic immune ageing. However, immune ageing can differ substantially across lineages and tissues [19,110,114]. T-cells, B-cells, NK-cells, and myeloid lineages can exhibit distinct age-associated burdens that vary across compartments (e.g., bone marrow, lymph nodes, mucosa, and peripheral blood). A practical direction is a hierarchical framework spanning cell-, tissue-, and individual-level axes.

At the cellular level, memory T-cell epigenetic clocks may not track host chronological age directly, but instead reflect division history and cumulative antigen exposure as an intrinsic timer. This motivates cell-type-specific clocks across major lineages (T-, B-, NK-, and myeloid cells) to localise which compartments show earlier or stronger ageing signals. At the tissue level, age-related alterations in the bone marrow niche and lymphoid architecture may exert lasting effects on systemic immune readouts via haematopoiesis, lymphatic drainage, and local antigen presentation. Cross-tissue transcriptomic resources and spatial-omics technologies can support tissue-specific immune clocks in sites such as the bone marrow, lymph nodes, spleen, and mucosa, clarifying how local ageing contributes to systemic immune ageing [71,129].

At the individual level, whole-blood transcriptional age and immune-specific clocks can provide both a global summary and complementary immune-focused readouts [14,71]. These measures enable cross-cohort comparisons and help distinguish immune ageing patterns from broader organismal ageing signals. Integrating the three axes may translate a generic “immune age gap” into more actionable statements, such as earlier ageing signals in specific T-cell lineages or in particular tissue niches (e.g., the bone marrow).

### 5.3. Intervention-Oriented Clocks and Causal Pathways

To move beyond correlational scoring, clocks should be linked to mechanisms and interventions. Prioritising stable, biologically coherent features (modules, pathways, cell-state programmes) facilitates interpretation and experimental follow-up. Integration with genetic variation, longitudinal designs and perturbation datasets (vaccination, infection, drug trials) can help separate causal drivers from downstream markers. Ultimately, a clinically useful immune ageing clock should support actionable decisions—who to intervene on, which pathway to target and whether the intervention modifies the underlying immune ageing trajectory.

Lifestyle factors and pharmacologic exposures can modulate inflammatory ageing trajectories. Sustained physical activity and improved circadian alignment have been associated with lower chronic low-grade inflammation and improved heart rate variability. mTORC1 inhibitors (e.g., rapalogs) have been reported to improve vaccine responsiveness and reduce respiratory infection risk in older adults. Senolytic regimens such as dasatinib plus quercetin (D + Q) have shown early signals consistent with a reduced senescence burden and functional improvement in initial studies [12]. To integrate clock readouts into intervention pathways, it is necessary to test whether clock changes translate into clinical benefits and to refine model weights toward modifiable lineages/pathways within a co-design loop supported by causal evidence [103,104,112].

### 5.4. Toward a Shared “Immune Age” Language

For clinical and public health adoption, a single strong model or validation in one cohort is insufficient. A key goal is a verifiable and recalibratable immunological age scale that is comparable across populations and settings. Building such a system requires at least three components. First, methodological rigour and end-to-end reporting traceability are essential. Predictive studies should ensure that data splitting, feature preparation, hyperparameter search, and internal/external validation are reproducible. For AI-assisted decision-making, studies should specify where the clock enters the decision pathway and evaluate whether its use improves outcomes or net benefits [31,59]. Second, discrimination, calibration and clinical net benefits should be reported jointly; reliance on a single favourable metric can overstate practical utility. Third, cross-cohort recalibration and transfer mechanisms are needed so that scores retain consistent meaning across groups, platforms, and contexts, enabling ongoing validation and updating [29,61,94].

Testing universality and transferability requires large-scale, multi-ethnic cohorts with cross-platform measurements and long-term follow-up (e.g., UK Biobank, All of Us, China Kadoorie Biobank, and FinnGen). Coordination with international immunome and atlas efforts can help map immune ageing across diverse genetic backgrounds, exposures, and disease contexts [129,130]. Within such frameworks, sharing feature definitions, model implementations, and recalibration scripts under harmonised metadata and reporting standards would support convergence toward comparable metrics.

Interpretation and communication require an immune age narrative that is coherent for both scientific and non-scientific audiences. A key challenge is to explain “accelerated immune ageing” without exaggerating risk, to link scores to modifiable factors where evidence exists, and to define appropriate guardrails for decision-making based on clock readouts [99], and making sure there are limits on changing strategies based on clock results within rules and paths, which need similar ways.

Ultimately, the value of immune ageing clocks will depend less on accumulating additional predictors than on building a quantitative language that withstands causal testing, supports comparability, and links to modifiable biology across cells, tissues, individuals, and timescales. This is the basis on which “immune age” could support precision prevention and individualised intervention rather than remaining a descriptive label [14,104].

## 6. Conclusions

Viewing prior studies through the three-axis task–modality–model framework clarifies the landscape of immune ageing clocks. From the task axis, existing approaches fall into three broad categories. Age clocks are supervised by chronological age; risk clocks are trained on clinical endpoints such as mortality or multimorbidity; and cell/lineage clocks emphasise immune lineages and adaptive receptor repertoires. Each captures distinct facets of age-related immune remodelling, and their outputs are not directly interchangeable. Across modalities, organising inputs into cell-layer and molecular-layer measurements, together with model families and evaluation criteria, yields an end-to-end methodological workflow. Taken together, the pipeline spans cohort and sample selection, data acquisition and standardisation, model training, validation, and recalibration, enabling clearer interpretation and cross-study comparability.

Despite rapid progress, several barriers limit robustness, comparability, and translation. Cohort heterogeneity and high-dimensional profiling amplify batch effects and compositional confounding. Models may inadvertently learn platform artefacts, technical noise, or composition shifts rather than biological ageing signals. Risk clocks trained on mortality or multimorbidity may perform well within a cohort, yet cross-cohort benchmarking and longitudinal recalibration are still uncommon. This limits its transferability and interpretability across different populations and situations. Moreover, many models rely on large feature sets whose links to testable immune mechanisms remain unclear. Risk characterisation and mechanism interpretation/intervention design are still separate. Without clearer task specification, principled modality selection, and standardised reporting, immune ageing clocks will remain difficult to reproduce and to deploy in practice.

Progress will depend less on marginal algorithmic gains than on aligning task–modality–model choices with specific decisions. Longitudinal designs and paired multi-omics can connect cross-sectional snapshots to trajectories and resolve lineage-specific ageing states. Digital phenotypes (vital signs, infection and vaccination history) may support immune digital twins that move clocks from static scores toward dynamic state estimates. To translate clocks, models should be anchored to testable pathways (inflammation, immunometabolism, clonal haematopoiesis) and evaluated within intervention frameworks. Practically, the field needs standardised, shareable pipelines: explicit task definitions, transparent data/feature provenance, rigorous external validation and documented recalibration. A minimal reproducibility package should include QC/preprocessing scripts, a frozen feature matrix with a data dictionary, model weights and hyperparameters, evaluation and recalibration code and an executable environment (e.g., Conda/Docker), linked to processed-data repositories.

## Figures and Tables

**Table 1:** Comparison of prior reviews and this review across key methodological dimensions for immune ageing clocks.

Dimension	Prior Reviews (Typical Coverage)	This Review (What We Add)
Tasks	Often mixed or implicit (age vs. outcome vs. lineage-level) [4,34]	Explicit task taxonomy: age/risk(outcome)/cell lineage (intracellular and repertoire) [13,14,35]
Modality	Described by assay types, less by analytical unit [36,37,38]	“Cell layer vs. molecular layer vs. clinical–digital context” as modelling units + cross-level mapping [25,39,40]
Models	Broad ML overview, limited linkage to task/modality [41,42]	Model choices mapped to task–modality constraints (statistics/ML/DL) [43,44]
Fusion	Mentioned but not systematised [45,46]	Early vs. late fusion as a design decision with pros/cons and failure modes [47,48,49]
Evaluation	Emphasis on fit/correlation/AUC [19,20]	Adds calibration, clinical net benefit, and deployment metrics as core [29,30,31]
Recalibration/transportability	Often brief or absent	Recalibration + internal–external CV/LOCO as standard practice [33,50]
Reporting	General reproducibility statements [51,52,53]	Concrete reporting checklist: leakage control, covariates, missingness, calibration, code/data share [54,55,56,57,58,59]

**Table 2:** Representative immune ageing clocks, immune risk scores, and related methodological frameworks.

Clock/Framework	Data Modality and Features	Population/Cohort	Primary Target/Outcome	Modelling Strategy	Key Message/Methodological Contribution
IMM-AGE [13]	Longitudinal high-dimensional immune phenotyping (flow cytometry panels, plasma cytokines), clinical covariates	~135 adults (8-year follow-up) from the Stanford 1000 Immunomes-related cohorts	Composite “immune age” capturing longitudinal immune remodelling; association with mortality, CMV status, inflammatory burden	Regularised regression and manifold learning on longitudinal immune features	Defines a clinically meaningful immune age metric derived from longitudinal immune system trajectories, integrating multiple immune cell subsets and soluble markers, and linking immune age acceleration to morbidity and mortality.
iAge—inflammatory ageing clock [14]	Deep profiling of plasma inflammatory proteome (“blood immunome”, >50 cytokines/chemokines), clinical phenotypes	1001 individuals aged 8–96 years, multi-cohort; centenarians included	Continuous “inflammatory age” (iAge); multimorbidity index, frailty, immunosenescence, cardiovascular ageing, exceptional longevity	Deep-learning model trained on inflammatory protein patterns; feature attribution to identify key drivers (e.g., CXCL9)	Establishes a deep-learning inflammatory ageing clock that tracks multimorbidity, frailty, and vascular ageing; demonstrates that CXCL9-driven vascular inflammation is a modifiable axis of inflammaging with mechanistic validation.
Immune Risk Profile (IRP)—Swedish NONA/OCTO studies [106]	Conventional immunophenotyping: CD4/CD8 ratio, CD8 + CD28– T-cells, naïve/memory subsets, CMV serostatus	Very old Swedish cohorts (OCTO/NONA), individuals in their 80 s–100 s	All-cause mortality and frailty in very old adults	Rule-based composite risk profile (CD4/CD8 < 1, expanded CD8 + CD28–, CMV+)	Classical immune risk score linking T-cell compartment remodelling and CMV-driven immunosenescence to short-term mortality; centenarians lacking the IRP suggest “escaped” immune risk despite extreme age.
IgG glycan “glycan age” clock [107]	LC-MS-based profiling of IgG N-glycans; 24 glycan traits summarising Fc glycosylation	>5000 individuals from multiple population cohorts	Chronological age, cardiometabolic risk and lifestyle factors; “glycan age” acceleration	Linear models and age-prediction regression using IgG glycan traits	Demonstrates that IgG glycan patterns constitute a sensitive biomarker of biological and chronological age; links pro-inflammatory glycan shifts to cardiometabolic risk, positioning humoral immune glycome as an immune-centric ageing clock.
PBMC scRNA-seq ageing clocks [73]	Single-cell RNA-seq of PBMCs; cell-type composition and gene expression; multi-cohort integration with supercentenarians	Multi-cohort ageing series plus 7 supercentenarians; adult age range plus extreme longevity	Cell composition and transcriptomic ageing clocks at a single-cell resolution; supercentenarian “biological age delay”	Partial least squares regression on cell-type composition; gene-expression-based clocks within major immune lineages	Constructs human PBMC single-cell immune ageing clocks, revealing that supercentenarians exhibit delayed immune ageing and a “ribosome–inflammation” equilibrium; highlights cell-type-specific transcriptional hallmarks of slow immune ageing.
sc-ImmuAging—cell-type-specific immune ageing clocks [108]	PBMC scRNA-seq (five large human cohorts); gene-level features in CD4, CD8, B-, NK-cells, and monocytes	1081 healthy European adults (18–97 years), plus external validation cohorts, COVID-19 and BCG vaccination cohorts	Cell-type-specific transcriptomic immune age; age acceleration/rejuvenation during infection and vaccination	Feature selection (correlation, mutual information, MIRA) + LASSO, random forest and PointNet deep-learning; separate clocks per cell type	Establishes robust cell-type-specific single-cell immune ageing clocks, reveals COVID-19-induced age acceleration (especially in monocytes) and identifies CD8+ T-cell age rejuvenation in individuals with strong interferon signatures after BCG vaccination.

**Table 3:** Methodological issues and representative tools for cross-cohort immune ageing clock development and deployment.

Methodological Issue	Tool/Method Category (Representative Tools)	Typical Use Scenarios	Strengths	Key Limitations/Risks (Explicitly Named in the Review)	Representative References (SCI/High-Impact Preferred)
Batch effects/platform heterogeneity	Empirical Bayes batch correction (ComBat)	Pre-integration correction for “sample-by-feature” matrices in bulk transcriptomics/proteomics/methylation across cohorts	Mature and widely validated; transparent statistical assumptions	Strong dependence on correct specification of batch and covariates; when true age-related biology is entangled with batch, genuine signals may be attenuated (“over-correction”); new external samples often require additional handling	Johnson et al., Biostatistics 2007 [84]
Batch effects/platform heterogeneity	Latent factor/surrogate-variable adjustment (SVA)	Unknown or incompletely annotated technical variation	Captures unobserved systematic noise and improves robustness	Can remove biology correlated with phenotype/age; requires careful design matrix specification	Leek et al., Bioinformatics 2012 [85]
Batch effects/platform heterogeneity	Removal of unwanted variation (RUV/RUVSeq)	RNA-seq with ERCC spike-ins, technical replicates, or definable negative control genes	Leverages controls; often more stable than naïve regression-only approaches	Mis-specified control sets introduce bias; risk of over-correcting genuine biological gradients	Risso et al., Nature Biotechnology 2014 [120]
Batch effects/platform heterogeneity (deployment-oriented)	Prediction-focused batch adjustment (fSVA)	Real-world deployment with single-sample or small-batch prediction (clinical/translational settings)	“Freezes” latent factors learned from training data for new-sample adjustment; closer to actual prediction pipelines	Depends on representativeness of the training cohort; limited benefit under strong target-domain drift	Parker et al., PeerJ 2014 [121]
Batch effects/platform heterogeneity (cytometry)	Control-based normalisation (CytoNorm)	Multi-batch flow/mass cytometry with instrument drift; bridge/control samples available	Highly practical for cytometry drift; emphasises control consistency	Requires high-quality controls and consistent gating/labelling; control design errors propagate bias	Van Gassen et al., Cytometry Part A 2020 [87]
Compositional confounding (bulk “mixtures”)	Partial deconvolution: proportion estimation (CIBERSORT)	Bulk tissue/whole blood/PBMC transcriptomics: estimate immune cell fractions	Classic, interpretable, broadly used	Limited transferability of signature matrices; unstable for rare subsets or strong inflammatory/therapy-driven compositional shifts	Newman et al., Nat. Biotechnol. 2019 [122]
Compositional confounding (bulk + multi-donor references)	Multi-subject scRNA reference deconvolution (MuSiC)	Bulk RNA-seq with multi-donor scRNA references; cross-population/cross-cohort studies	Uses cross-donor stable markers; often more robust across populations	Biassed when reference and target tissue/state mismatch; sensitive to novel states/subtypes	Wang et al., Nature Communications 2019 [90]
Compositional confounding (bulk + scRNA reference)	Reference-based decomposition models (BisqueRNA)	Bulk RNA-seq with scRNA reference; relatively stable cell-type labels available	Clear engineering workflow; practical for routine cohorts	Depends on annotation and depth comparability; larger errors for rare populations	Jew et al., Nature Communications 2020 [118]
Compositional confounding (method selection)	Benchmarking and comparative evaluations	Supporting a “do not rely on a single algorithm” recommendation in the review	Provides empirical determinants: preprocessing, marker choice, composition, method sensitivity	Conclusions are tissue- and dataset-dependent; cannot be blindly generalised	Avila Cobos et al., Nature Communications 2020 [91]
Compositional confounding (epigenomics)	Cell-mixture adjustment (reference-based) (Houseman method)	Whole-blood DNA methylation (DNAm): mixture effects strongly distort age/disease signals	One of the standard approaches in DNAm mixture correction; can be cross-validated with cytometry	Bias under reference mismatch across populations/platforms; incomplete reference panels leave residual confounding	Houseman et al., BMC Bioinformatics 2012 [18]

## Data Availability

No new data were created or analysed in this study.
